# Letter to the Editor: Financial incentives for COVID-19 vaccination

**DOI:** 10.4178/epih.e2021088

**Published:** 2021-10-22

**Authors:** Hyuncheol Bryant Kim

**Affiliations:** Department of Economics and Division of Public Policy, Hong Kong University of Science and Technology, Hong Kong

Dear Editor,

The coronavirus disease 2019 (COVID-19) vaccine is the most important weapon in the fight against the COVID-19 pandemic. Vaccination not only lowers the chances of COVID-19 infection, but also significantly reduces the risk of hospitalization and death from COVID-19 [1,2]. Due to the Delta variant, the scenario of ending the COVID-19 pandemic through herd immunity is no longer an option [3]. To live with COVID-19 is inevitable. A suppression strategy including intensive social distancing, contact tracing, and border control cannot last forever. Many countries, including the United Kingdom, Israel, and Singapore, have already moved toward a new normal life coexisting with COVID-19. Vaccine take-up rates, especially for the elderly, are expected to play a critical role in determining the number of infections and hospitalizations, and preventing a collapse of the health system.

There are three main approaches to promote vaccine take-up: (1) information provision and persuasion, (2) financial incentives, and (3) non-financial incentives (e.g., vaccine passes, travel restrictions, etc.). Financial incentives (or subsidies) promote vaccine take-up by reducing the cost of vaccination. Although vaccines are free in most countries, people have to bear indirect costs, which include costs related to transportation and foregone income, as well as psychological and monetary costs that may be caused by the side effects of vaccination. Indeed, a large-scale randomized controlled trial in Sweden showed that modest financial incentives (US$24≈SEK 200) increased the COVID-19 vaccine take-up rate by 4.2 percentage points, from a baseline rate of 71.6% [4].

Many countries have already introduced different types of financial incentives, including vaccination lotteries and conditional lump-sum transfers. For example, in the United States, New York, Ohio, and California provided a lottery to those who got vaccinated. In contrast, West Virginia, Maryland, and Detroit paid a fixed amount of cash (between US$50 and 100). Similarly, Greece offered 150 Euros for young adults aged between 18 and 25, and Serbia provided 25 Euros for all age groups as vaccine incentives. In some countries, private companies have introduced a lottery for those vaccinated. Sino Group, a Hong Kong real estate company, provided a one-room apartment worth about US$1.3 million through a lottery among vaccinated people. Qantas Airways in Australia has announced that it will offer ticket discounts for those who have been vaccinated.

Some authors have opposed paying people to get vaccinated, arguing that financial incentives are immoral, ineffective, and coercive [5]. However, these arguments are illogical and misleading [6]. Financial incentives to compensate for time and lost income are morally appropriate and effectively increase vaccine take-up [4]. Furthermore, it is inappropriate to argue that financial incentives are simply coercive. How people feel about these incentives depends on how society offers them.

An important question is how to design a financial incentive. For example, is a vaccine lottery or a conditional lump-sum transfer better as a vaccine incentive? Suppose that there are two groups of people that consist of 100,000 people. The first group could receive a US$100 cash incentive for vaccination, and the second group could receive a lottery that pays US$1 million with 0.01% odds. The expected value for those in both groups is US$100. Prospect theory predicts that the lottery could be better [7]. People generally tend to overestimate very low probabilities, which is called ‘probability distortion.’ This phenomenon explains why people buy lottery tickets even though doing so is, on average, a loss. People would value the lottery greater than the cash with the same expected value. A recent laboratory experiment study indeed showed that a lottery could improve COVID-19 vaccine take-up more than a lump-sum transfer, although the study was based on a laboratory experiment under hypothetical settings [8].

In addition, lotteries could have stronger spillover effects than lump-sum payments because lottery winners of large amounts of money often receive media coverage. This exposure could increase vaccine take-up even further. It is well known that peer effects could play an important role in the adoption of health products and technology [9]. Due to the probability distortion and peer effects, the lottery could be more effective for promoting vaccine take-up. However, research is needed to test this prediction empirically.

Lastly, I would like to point out that the financial incentives for COVID-19 are temporary. Although temporary financial incentives have been shown to encourage vaccine take-up in the short run, this could have unintended consequences in the long run. The long-term effects are potentially ambiguous in theory. Temporary subsidies can reduce take-up in the long run if people consider the temporarily subsidized price as a reference point that could affect their future reservation price [10]. A financial incentive could decrease vaccine uptake in the future when it disappears. However, temporary subsidies can increase demand in the long run if people experience minor or no side effects and learn the value and benefits of vaccination. Despite the importance of understanding the long-term effects of temporary subsidies, there is relatively scarce evidence on this topic. Future research is needed to provide evidence on the long-term effects of financial incentives.
